# Impact of histological subtype on prognosis in stage IV colorectal cancer: A population-based cohort study

**DOI:** 10.1371/journal.pone.0264652

**Published:** 2022-03-03

**Authors:** Teppei Miyakawa, Hidetaka Kawamura, Michitaka Honda, Yoshinao Takano, Shunji Kinuta, Takahiro Kamiga, Shigeru Yamazaki, Atsushi Muto, Satoru Shiraso, Naoyuki Yamashita, Toshiyasu Iwao, Koji Kono, Shinichi Konno

**Affiliations:** 1 Department of Minimally Invasive Surgical and Medical Oncology, Fukushima Medical University, Fukushima, Fukushima, Japan; 2 Department of Surgical Oncology, Southern Tohoku General Hospital, Southern Tohoku Research Institute for Neuroscience, Koriyama, Fukushima, Japan; 3 Department of Surgery, The Takeda Healthcare Foundation Takeda General Hospital, Aizu Wakamatsu, Fukushima, Japan; 4 Department of Surgery, Shirakawa Kosei General Hospital, Shirakawa, Fukushima, Japan; 5 Department of Surgery, Ohta Nishinouchi Hospital, Koriyama, Fukushima, Japan; 6 Department of Surgery, Fukushima Rosai Hospital, Iwaki, Fukushima, Japan; 7 Department of Surgery, Iwaki Kyoritsu General Hospital, Iwaki, Fukushima, Japan; 8 Department of Surgery, Tsuboi Hospital, Koriyama, Fukushima, Japan; 9 Department of Gastroenterology, Aidu Chuo Hospital, Aizu Wakamatsu, Fukushima, Japan; 10 Department of Gastrointestinal Tract Surgery, Fukushima Medical University, Fukushima, Fukushima, Japan; 11 Department of Orthopedic Surgery, School Medicine, Fukushima Medical University, Fukushima, Japan; Chang Gung Memorial Hospital and Chang Gung University, Taoyuan, Taiwan, TAIWAN

## Abstract

**Background:**

There are a few established prognostic factors for stage IV colorectal cancer. Thus, this study aimed to evaluate the impact of histological subtypes on prognosis and metastatic patterns in patients with stage IV colorectal cancer.

**Methods:**

This was a population-based, multicenter, cohort study. We included consecutive patients diagnosed with stage IV colorectal cancer between 2008 and 2015 at all designated cancer hospitals in Fukushima prefecture, Japan. Patients were classified into two groups according to histological subtypes as follows: poorly differentiated adenocarcinoma (Por), mucinous adenocarcinoma (Muc), or signet-ring cell carcinoma (Sig) and well (Wel) or moderately differentiated adenocarcinoma (Mod). We evaluated the relationship between these histological groups and survival time. After adjusting for other clinical factors, we calculated the hazard ratio for Por/Muc/Sig.

**Results:**

A total of 1,151 patients were enrolled, and 1,031 and 120 had Wel/Mod and Por/Muc/Sig, respectively. The median overall survival was 19.2 and 11.9 months for Wel/Mod and Por/Muc/Sig, respectively (p < 0.001). The adjusted hazard ratio for Por/Muc/Sig with regard to survival time was 1.42 (95% confidence interval: 1.13–1.77). Por/Muc/Sig had a lower incidence of liver and lung metastases and a higher incidence of peritoneal dissemination and metastasis to rare organs, such as the bone and brain.

**Conclusions:**

The Por/Muc/Sig histological subtype was an independent prognostic factor for poor prognosis among patients with stage IV colorectal cancer. The histological subtype may be useful for predicting the prognosis of patients with stage IV colorectal cancer and designing the treatment strategy.

## Introduction

Although recent advances in systemic chemotherapy, including targeted molecular agents, have contributed to the improvement of survival rates, the prognosis of stage IV colorectal cancer (CRC) with distant metastasis remains poor [1]. Both primary and metastatic sites need to be considered in the treatment strategy, and in many cases, multiple surgeries or long-term chemotherapy are required. In cases where cure may be infeasible, it is important to preserve the patient’s quality of life and to maintain a balance between the benefits and adverse events of treatment. Therefore, it is crucial to determine individual prognoses as accurately as possible when deciding on a treatment strategy for stage IV CRC.

It is clinically important to identify prognostic factors of stage IV CRC that can be easily used in general practice, such as the histological subtype of biopsy tissue. Most CRCs are differentiated adenocarcinomas, but approximately 10% are poorly differentiated adenocarcinoma (Por), 10% are mucinous carcinoma (Muc), and 1% are signet-ring cell carcinomas (Sig) [2–4]. Por/Muc/Sig CRC is genetically known to have a high incidence of deficient mismatch repair (dMMR), which causes microsatellite instability (MSI) and *BRAF* mutations [2, 4–7]. These genetic statuses are associated with a poor prognosis in stage IV CRC [8]. Thus, histological subtype may be used as a prognostic factor without the need for such genetic analysis.

We hypothesized that histological subtype based on the biopsy of the primary tumor could be a prognostic factor for stage IV CRC. We examined the prognostic impact of histological subtypes using data from a large cohort. In this cohort, two gastrointestinal surgeons, who were blinded to the survival outcome, reviewed the medical records and computed tomography (CT) images before initial treatment and made the diagnosis based on the 7th edition of TNM classification. Unlike stage I/II/III, in which detailed pathological diagnosis can be obtained from surgically resected specimens, there are a few established prognostic factors in stage IV CRC. The identification of prognostic factors in stage IV CRC that can be easily used in clinical practice would provide meaningful information to clinicians.

## Materials and methods

### Study design and cohort development

This was a historical population-based cohort study. All nine designated cancer hospitals across Fukushima prefecture participated in this study. First, we extracted the data of patients with stage IV CRC from hospital-based cancer registries. Second, we extracted the patients’ data, including Charlson comorbidity index (CCI), clinical symptoms, cTNM stage (based on the 7th edition of TNM classification), primary tumor site, Barthel index (as a measure of the activity of daily living), presence of primary tumor resection, and treatment for metastatic sites (metastasectomy, doublet chemotherapy, or best supportive care [BSC]). Two gastrointestinal surgeons (MH and HK), who were blinded to the survival outcome, reviewed the medical records and computed tomography (CT) images before initial treatment in this cohort and made the diagnosis based on cTNM staging, metastatic pattern. Doublet chemotherapy was defined as two or more courses of doublet chemotherapy, including oxaliplatin or irinotecan. We defined BSC as the non-use of metastasectomy or chemotherapy. Finally, we merged the datasets from each participating institute after anonymizing the information.

We enrolled consecutive adult patients (≥ 18 years old) with histologically confirmed colorectal adenocarcinoma who were clinically or intraoperatively diagnosed with stage IV CRC (International Classification of Diseases, Tenth Revision codes, C18, C19, and C20) between 2008 and 2015. Patients who lacked data on the histological subtype or treatment type, or who were lost to follow-up were excluded.

### Ethics approval and consent to participate

This study was conducted in accordance with the Declaration of Helsinki and relevant local laws and regulations. The approval for the protocol was obtained from the institutional review board of all the participating hospitals (Southern Tohoku General Hospital, The Takeda Healthcare Foundation Takeda General Hospital, Shirakawa Kosei General Hospital, Ohta Nishinouchi Hospital, Fukushima Rosai Hospital, Iwaki Kyoritsu General Hospital, Tsuboi Hospital, Aidu Chuo Hospital, and Fukushima Medical University), and the study protocol was registered in the UMIN-CTR Clinical Trial registry, a priori (UMIN000033718). The anonymous nature of the data allowed the requirement for informed consent to be waived.

### Histological subtype

The histological subtype was classified as follows: Wel/Mod group (well or moderately differentiated adenocarcinoma) and Por/Muc/Sig group. We diagnosed the histological type when it is a quantitatively dominant tissue type.

### Statistical analysis

The primary outcome was the adjusted hazard ratio (HR) of each histological subtype group for overall survival (OS), calculated as the number of days from the date of CRC diagnosis until death or December 31, 2018. Patients who had not experienced any events of interest were censored at the date of the final observation. To compare the OS by histological group, we evaluated the descriptive statistics and extracted age, sex, CCI, primary tumor site, T-factor, N-factor, liver metastasis, lung metastasis, peritoneal dissemination, distal lymph node metastasis, other-organ metastasis, and the number of organs with metastasis as potential confounding factors at the consensus meeting. After adjusting for these factors, we calculated the HR and 95% confidence interval (95% CI) of each histological subtype using the Cox proportional-hazards model. In addition, we evaluated the survival curve for all patients and three subgroups as follows: patients that were administered 1) metastasectomy, 2) doublet chemotherapy, or 3) BSC. We used the Kaplan-Meier method for each histological group and performed the log-rank test. Moreover, we performed a multivariate analysis as a subgroup analysis for each treatment and calculated the adjusted HR of each histological subtype using the Cox proportional-hazards model.

The secondary outcome included metastatic patterns. Based on the Japanese Classification of Colorectal Carcinoma [9], we distinguished the following types of metastases: liver metastasis (H1, ≤5 hepatic tumors [HTs] and HT size ≤5 cm; H2, ≥5 HTs or HT size ≥5 cm; and H3, ≥5 HTs and HT size ≥5 cm), and pulmonary metastasis (PUL1, <3 lung tumors [LTs] in one lung, or two LTs in both lungs; PUL2, ≥3 LTs in both lungs, carcinomatous pleurisy, or mediastinum lymph node metastasis).

Patient characteristics were reported as descriptive statistics; continuous variables are expressed as medians and interquartile ranges (IQRs), and categorical variables are expressed as counts and percentages. Univariate analyses were used to compare patient characteristics between the two histological groups. Categorical variables were compared using the chi-square test, and continuous variables were compared using the Student t-test. Survival analysis was performed using the Kaplan-Meier method, and survival estimates were compared using the log-rank test. The threshold for significance was a p-value of <0.05. All data management and statistical analyses were conducted using STATA version 16 (StataCorp, College Station, TX, USA).

## Results

**Fig 1:**
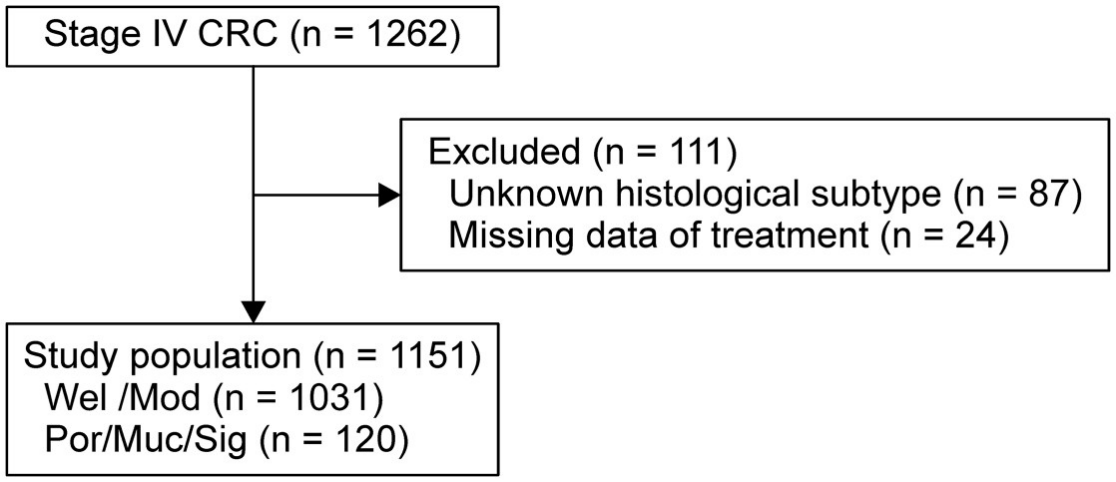
Patient enrollment flow.

Fig 1 shows the patient enrollment flow. A total of 1262 patients with stage IV CRC were identified from the databases, and 1151 patients were enrolled in this study. Table 1 shows the patient characteristics. There were 1031 (90%) and 120 (10%) patients in the Wel/Mod and Por/Muc/Sig groups, respectively. There were no significant differences in age, sex, CCI, Barthel index, and symptoms of primary site between the two groups. The Por/Muc/Sig group had a higher proportion of RCC as primary tumor site compared to the Wel/Mod group [67 (56%) and 340 (33%), respectively]. The incidences of T-stage and N-stage in the Wel/Mod and Por/Muc/Sig groups were as follows: T4b stage (Wel/Mod: 197 [19%], Por/Muc/Sig: 32 [27%]); N2 stage (Wel/Mod: 515 [50%], Por/Muc/Sig: 78 [65%]). In the Por/Muc/Sig group, the proportion of patients who treated with the doublet chemotherapy (Wel/Mod: 561 [54%], Por/Muc/Sig: 54 [45%]) and metastasectomy (Wel/Mod: 200 [19%], Por/Muc/Sig: 12 [10%]) was low, and the BSC (Wel/Mod: 235 [23%], Por/Muc/Sig: 43 [36%]) was high.

**Table 1:** Patients’ characteristics.

	Wel/Mod	Por/Muc/Sig	*p* value
	(n = 1031)	(n = 120)	
**Age in years, median (IQR)**	69 (66–77)	70 (60–77)	0.68
**Sex, n (%)**			
Male	646 (62.7%)	65 (54.2%)	0.070
Female	385 (37.3%)	55 (45.8%)	
**CCI, n (%)**			
0	534 (51.8%)	76 (63.3%)	0.055
1, 2	393 (38.1%)	34 (28.3%)	
≥3	104 (10.1%)	10 (8.3%)	
**Barthel index, n (%)**			
100	679 (65.9%)	74 (61.7%)	0.34
99–91	12 (1.2%)	0 (0.0%)	
90–61	68 (6.6%)	13 (10.8%)	
60–21	67 (6.5%)	8 (6.7%)	
20–0	66 (6.4%)	7 (5.8%)	
Unknown	139 (13.5%)	18 (15.0%)	
**Symptom of primary site, n (%)**			
None	380 (36.9%)	49 (40.8%)	0.39
Obstruction	564 (54.7%)	59 (49.2%)	0.36
Bleeding	112 (10.9%)	12 (10.0%)	0.76
Perforation	34 (3.3%)	6 (5.0%)	0.53
**Primary site, n (%)**			
RCC	340 (33.0%)	67 (55.8%)	<0.001
LCC	435 (42.2%)	29 (24.2%)	
Rectal cancer	242 (23.5%)	21 (17.5%)	
Unknown	14 (1.4%)	3 (2.5%)	
**T-stage, n (%)**			
T1・T2	32 (3.1%)	1 (0.8%)	0.025
T3	330 (32.0%)	26 (21.7%)	
T4a	472 (45.8%)	61 (50.8%)	
T4b	197 (19.1%)	32 (26.7%)	
**N-stage, n (%)**			
N0	164 (15.9%)	5 (4.2%)	<0.001
N1	352 (34.1%)	37 (30.8%)	
N2	515 (50.0%)	78 (65.0%)	
**Number of metastatic organ, n (%)**			
1	629 (61.0%)	60 (50.0%)	0.066
2	264 (25.6%)	39 (32.5%)	
≥3	138 (13.4%)	21 (17.5%)	
**Primary tumor resection, n (%)**	693 (67.2%)	78 (65.0%)	0.63
**Metastasectomy, n (%)**	200 (19.4%)	12 (10.0%)	0.006
**1st line Chemotherapy, n (%)**			
Total	778 (75.5%)	75 (62.5%)	0.002
Doublet chemotherapy	561 (54.4%)	54 (45.0%)	0.032
Cytotoxic agents			
Oxaliplatine	538 (52.2%)	52 (43.3%)	0.15
Irinotecan	23 (2.2%)	2 (1.7%)	
Molecular targeted therapy			
None	236 (22.9%)	19 (15.8%)	0.86
VEGF	282 (27.3%)	30 (25.0%)	
EGFR	43 (4.2%)	5 (4.2%)	
**2nd line Chemotherapy, n (%)**			
Total	543 (52.7%)	50 (41.7%)	0.026
Cytotoxic agents			
Others	114 (11.1%)	11 (9.2%)	0.17
Oxaliplatine	176 (17.1%)	15 (12.5%)	
Irinotecan	253 (24.5%)	24 (20.0%)	
**3rd line Chemotherapy, n (%)**			
Total	319 (30.9%)	28 (23.3%)	0.09
Cytotoxic agents			
Others	99 (9.6%)	5 (4.2%)	0.19
Oxaliplatine	71 (6.9%)	7 (5.8%)	
Irinotecan	149 (14.4%)	16 (13.3%)	
**1st line period of doublet chemotherapy, days, median (IQR)**	148 (73–235)	135.5 (70–248)	0.55
**Best supportive care, n (%)**	235 (22.8%)	43 (35.8%)	0.002

Abbreviations: IQR = interquartile range; CCI = charlson comorbidity index; RCC = right colon cancer; LCC = left colon cancer; VEGF = vascular endothelial growth factor; EGFR = epidermal growth factor receptor.

### Adjusted HRs and overall survival curves

The median follow-up time was 18.0 (IQR, 6.5–32.7) months, and 858 patients (75%) died during the study period. Table 2 shows the adjusted HRs for all patients. The adjusted HR (95% CI) was 1.42 (1.13–1.77) for the Por/Muc/Sig group compared to the Wel/Mod group. Adjusted HR for the T4b compared with the T1,2 and N2 compared with N0 were 1.17 (95% CI: 0.75–1.83) and 1.26 (95% CI: 1.01–1.57), respectively. Adjusted HR for the H1, H2, and H3 compared with the H0 were 0.89 (95% CI: 0.67–1.18), 1.81 (95% CI: 1.36–2.41), and 2.45 (95% CI: 1.84–3.27), respectively. Adjusted HR for the presence of peritoneal dissemination and other organ metastasis such as brain and bone were 1.41 (95% CI: 1.09–1.81) and 1.49 (95% CI: 1.07–2.07), respectively.

**Table 2:** Adjusted hazard ratio for overall survival in all patients.

	Unadjusted			Adjusted		
	HR	95% CI	*p* value	HR	95% CI	*p* value
**Differentiation**						
Wel/Mod	(reference)			(reference)		
Por/Muc/Sig	1.66	(1.35–2.04)	<0.001	1.42	(1.13–1.77)	0.002
**Age**						
<65	(reference)			(reference)		
65–74	1.11	(0.94–1.31)	0.22	1.14	(0.96–1.36)	0.14
≥75	1.60	(1.36–1.88)	<0.001	1.83	(1.54–2.18)	<0.001
**Sex**						
Male	(reference)			(reference)		
Female	1.11	(0.96–1.27)	0.16	0.99	(0.85–1.14)	0.85
**CCI**						
0	(reference)			(reference)		
1,2	1.07	(0.93–1.24)	0.34	1.06	(0.91–1.24)	0.46
≥3	1.36	(1.08–1.72)	0.008	1.18	(0.93–1.51)	0.18
**Primary site**						
RCC	(reference)			(reference)		
LCC	0.83	(0.71–0.97)	0.017	0.85	(0.72–1.00)	0.057
Rectal cancer	0.76	(0.64–0.92)	0.004	0.83	(0.68–1.01)	0.063
**T-stage**						
T1,2	(reference)			(reference)		
T3	0.84	(0.55–1.29)	0.42	0.76	(0.49–1.18)	0.22
T4a	1.13	(0.74–1.72)	0.56	0.91	(0.59–1.39)	0.65
T4b	1.40	(0.90–2.15)	0.13	1.17	(0.75–1.83)	0.50
**N-stage**						
N0	(reference)			(reference)		
N1	0.98	(0.79–1.21)	0.83	0.98	(0.79–1.22)	0.87
N2	1.47	(1.20–1.80)	<0.001	1.26	(1.01–1.57)	0.04
**Liver metastasis**						
H0	(reference)			(reference)		
H1	0.76	(0.63–0.92)	0.005	0.89	(0.67–1.18)	0.43
H2	1.51	(1.26–1.82)	<0.001	1.81	(1.36–2.41)	<0.001
H3	2.09	(1.73–2.53)	<0.001	2.45	(1.84–3.27)	<0.001
**Lung metastasis**						
PUL0	(reference)			(reference)		
PUL1	0.92	(0.71–1.19)	0.52	0.92	(0.66–1.29)	0.63
PUL2	1.46	(1.23–1.72)	<0.001	1.22	(0.93–1.61)	0.15
**Peritoneal dissemination**						
Absence	(reference)			(reference)		
Presence	1.51	(1.30–1.74)	<0.001	1.41	(1.09–1.81)	0.008
**Distal lymph node metastasis**						
Absence	(reference)			(reference)		
Presence	1.42	(1.22–1.65)	<0.001	1.15	(0.90–1.47)	0.27
**Other organ metastasis**						
Absence	(reference)			(reference)		
Presence	1.60	(1.25–2.05)	<0.001	1.49	(1.07–2.07)	0.018
**Number of metastatic organ**						
1	(reference)			(reference)		
2	1.58	(1.35–1.84)	<0.001	1.12	(0.86–1.46)	0.39
≥3	2.69	(2.22–3.26)	<0.001	1.44	(0.85–2.44)	0.17

Abbreviations: RCC = right colon cancer; LCC = left colon cancer; CCI = Charlson comorbidity index; CI = confidence interval; HR = hazard ratio.

**Fig 2:**
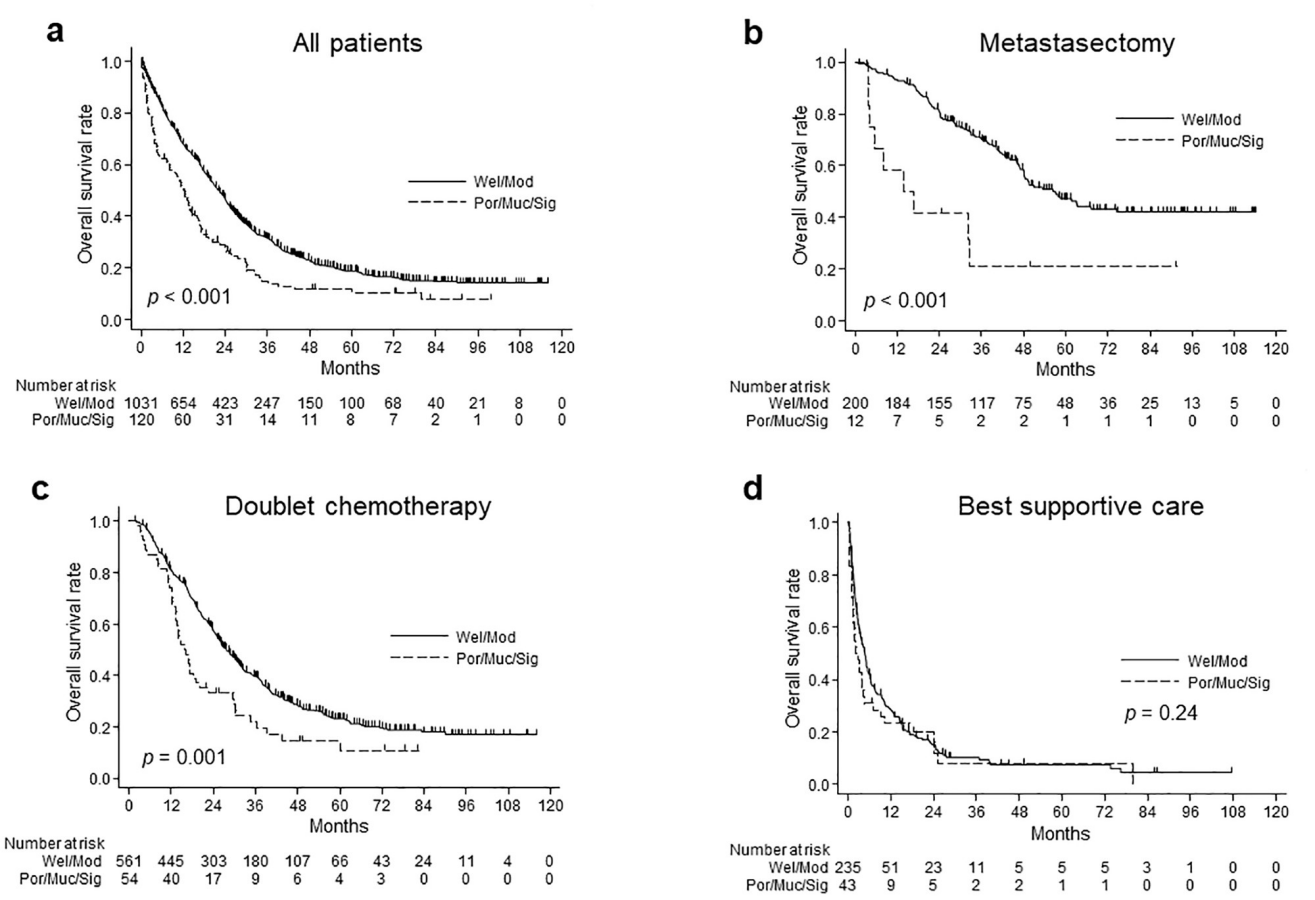
Overall survival according to histological subtype. a) All patients b) Patients with metastasectomy c) Patients with doublet chemotherapy d) Patients with best supportive care.

Fig 2 shows the OS rate and at-risk population for each histological group for all patients (Fig 2a), patients with metastasectomy (Fig 2b), patients with doublet chemotherapy (Fig 2c), and patients with BSC (Fig 2d). The median OS value for the Wel/Mod and Por/Muc/Sig groups was 19.2 and 11.9 months, respectively, (p < 0.001) for all patients, 40.9 and 15.3 months, respectively, (p < 0.001) for patients with metastasectomy, 25.8 and 16.1 months, respectively, (p = 0.001) for patients with doublet chemotherapy, and 3.3 and 2.2 months, respectively, (p = 0.24) for patients with BSC. Table 3 shows the adjusted HRs for the Por/Muc/Sig group compared to the Wel/Mod group in each treatment. The adjusted HR (95% CI) for patients with BSC and with doublet chemotherapy or metastasectomy was 1.02 (0.66–1.60) and 1.51 (1.10–2.06), respectively.

**Table 3:** Adjusted hazard ratio for overall survival in each treatment.

	Best supportive care			Doublet chemotherapy			Metastasectomy			Doublet chemotherapy or Metastasectomy		
	HR	95% CI	*p* value	HR	95% CI	*p* value	HR	95% CI	*p* value	HR	95% CI	*p* value
**Differentiation**												
Wel/Mod	(reference)			(reference)			(reference)			(reference)		
Por/Muc/Sig	1.02	(0.66–1.60)	0.91	1.48	(1.05–2.07)	0.023	2.25	(0.92–5.54)	0.077	1.51	(1.10–2.06)	0.011

Adjusted for age, sex, CCI, primary tumor site, T-factor, N-factor, liver metastasis, lung metastasis, peritoneal dissemination, distal lymph node metastasis, other-organ metastasis, and the number of organs with metastasis.

Abbreviations: HR = hazard ratio.

### Secondary outcomes

Table 4 shows details of the metastatic patterns. The Por/Muc/Sig group had a higher proportion of peritoneal dissemination compared to the Wel/Mod group [64 (53%) and 256 (25%), respectively (p < 0.001)]. In the Por/Muc/Sig group, 18 patients (15%) had metastases to other organs. Nine patients (7.5%) had bone metastases, three patients (2.5%) had brain metastases, three patients (2.5%) had skin metastases, and one patient each had pancreas, spleen, and ovary metastases. Bone and brain metastases were more frequent in the Por/Muc/Sig group.

**Table 4:** Metastatic pattern.

	Wel/Mod	Por/Muc/Sig	*p* value
	(n = 1031)	(n = 120)	
**Liver metastasis, n (%)**			
H0	282 (27.3%)	59 (49.2%)	<0.001
H1	278 (27.0%)	28 (23.3%)	
H2	247 (24.0%)	17 (14.2%)	
H3	224 (21.7%)	16 (13.2%)	
**Lung metastasis, n (%)**			
PUL0	733 (71.1%)	100 (83.3%)	0.016
PUL1	92 (8.9%)	5 (4.2%)	
PUL2	206 (20.0%)	15 (12.5%)	
**Peritoneal dissemination, n (%)**			
Absence	775 (75.2%)	56 (46.7%)	<0.001
Presence	256 (24.8%)	64 (53.3%)	
**Distal lymph node metastasis, n (%)**			
Absence	790 (76.6%)	74 (61.7%)	<0.001
Presence	241 (23.4%)	46 (38.3%)	
**Other organ metastasis, n (%)**			
Absence	964 (93.5%)	102 (85.0%)	0.001
Presence	67 (6.5%)	18 (15.0%)	
Bone	29 (2.8%)	9 (7.5%)	0.007
Brain	7 (0.7%)	3 (2.5%)	0.042

## Discussion

This study had two main findings. First, histological subtype was an independent prognostic factor in stage IV CRC after adjustment for age, sex, CCI, T-factor, N-factor, primary tumor site, number of metastatic organs, and location and severity of metastatic sites. Second, the metastatic pattern in stage IV CRC was influenced by the histological subtype. A previous study from Japan revealed an association between histological subtype and prognosis in stage IV CRC patients [10]. A previous study reported that adjusted HR for the Por/Muc/Sig group compared to the Wel/Mod group was 1.56(95% CI: 1.10–2.17), which demonstrated similar results to those found in our study. The previous study included patients with stage IV CRC who had undergone curative resection between 1997 and 2007 and were diagnosed based on the 6th edition of TNM, which lacks T4b (only T4, T4a, and T4b were introduced in the 7th edition). We believe that the novelty of our study compared to that of the previous study is in the following two points. First, all patients diagnosed with stage IV CRC, not only those who had undergone curative resection, were included in our study. Second, our study classified the severity of liver and lung metastases before treatment using CT scans, based on the Japanese Classification of Colorectal Carcinoma, and investigated T and N factors with CT scans before a treatment based on the 7th edition of TNM classification. To our knowledge, this is the first retrospective cohort study that evaluates the impact of histological subtype on prognosis using a dataset that included all patients with IV CRC, regardless of treatment status, after adjusting for confounding factors, such as the severity of the primary tumor and metastatic lesion (based on the 7th edition of TNM classification and Japanese Classification of Colorectal Carcinoma).

Previous studies reported that approximately 10% are poorly differentiated adenocarcinoma (Por), 10% are mucinous carcinoma (Muc), and 1% are signet-ring cell carcinomas (Sig) [2–4]. In this study, the distribution of the Por/Muc/Sig group was 10% and lower than previous studies reported. However, another retrospective study in Japan [10] reported that the distribution of the Por/Muc/Sig group of patients with stage IV CRC who underwent curative resection was 8% which was lower than that reported in this study. Considering the low proportion of metastasectomy in Por/Muc/Sig, the proportion of Por/Muc/Sig group (10%) in this study seems reasonable.

The proportion of patients who treated with the doublet chemotherapy and metastasectomy was low, and the BSC was high in the Por/Muc/Sig group. This may be due to diagnosis at a more advanced state or the presence of a lower performance status at diagnosis. Subgroup analysis showed no difference in the survival curve and multivariate analysis in patients with BSC, but in patients who underwent metastasectomy or doublet chemotherapy, Por/Muc/Sig had a worse prognosis. This suggests that the response to treatment was worse in the Por/Muc/Sig group. A retrospective study indicated that patients with Por/Muc/Sig CRC had a poor prognosis, even after curative metastasectomy [10]. They reported that the Por/Muc/Sig group had a higher incidence of peritoneal dissemination recurrence and multiple organ recurrence, and a lower rate of resection recurrence. In addition, other previous report also suggested that Por CRC was resistant to chemotherapy in the adjuvant setting [11]. Although the mechanisms underlying the chemotherapy resistance of Por CRC remain unclear, it has been reported that the activity of dihydropyrimidine dehydrogenase, a key metabolic enzyme of 5-fluorouracil, is higher in Por CRC [12]. High activity of dihydropyrimidine dehydrogenase is known to be a cause of resistance to 5-fluorouracil-based chemotherapy [13]. Moreover, several previous studies have suggested that Muc CRC is chemoresistant not only to 5-fluorouracil but also to systemic chemotherapy with oxaliplatin or irinotecan [14–17].

As noted in a previous study, Por/Muc/Sig had a lower incidence of liver and lung metastases and a higher incidence of peritoneal dissemination and distant lymph node metastases [10, 18]. In particular, peritoneal dissemination was observed in more than half of the cases in the Por/Muc/Sig group. Multiple organ metastases and metastasis to rare organs, such as the bone and brain, were also more likely to occur in the Por/Muc/Sig group. Early detection of uncommon and difficult-to-detect metastatic lesions such as peritoneal dissemination and bone and brain metastases may allow for more treatment options, including radical resection, and may contribute to prolonged survival. In addition to regular computed tomography (CT) scan, other modalities such as staging laparoscopy, head CT, positron emission tomography-CT, and magnetic resonance imaging may be considered. As metastases progress, they may cause intestinal obstruction, neurological symptoms, pain, pathological fractures, spinal nerve compression, and hypercalcemia, leading to a decrease in patients’ quality of life [19–21]. The early detection of these metastases may also contribute to early therapeutic intervention before symptoms develop, and close follow-up can be designed to maintain the patient’s quality of life. In addition, peritoneal dissemination, bone metastasis, and brain metastasis are known to have metastatic patterns that are associated with poor prognoses [19–23]. If curative metastasectomy is infeasible, it may be necessary to consider treatment options other than standard systemic chemotherapy. For example, in peritoneal dissemination, a recent randomized controlled trial suggested that cytoreductive surgery may have survival benefits [24]. Bone and brain metastases often require radiotherapy for local control, in addition to systemic chemotherapy.

We showed that the frequencies of T4b and N2 were higher in the Por/Muc/Sig group. These clinicopathological features of patients with Por/Muc/Sig CRC are consistent with those of previous studies [10, 25]. In addition, previous studies have reported that genetic and epigenetic statuses, such as dMMR/MSI and *BRAF* mutations, were more frequent in Por/Muc/Sig [2, 4–7]. dMMR/MSI and *BRAF* mutations are known risk factors for poor prognosis in stage IV CRC [8]. Thus, overall, patients with Por/Muc/Sig CRC may have metastatic patterns, clinicopathological features, and genetic and epigenetic statuses that are associated with a poorer prognosis.

One of the strengths of this study was that we included potentially important data such as comorbidities, T-factor, N-factor, or metastatic pattern and severity, because of the involvement of clinicians who extracted information from medical records and cross-validated it against imaging findings. Using these data, we could adjust for confounders. Another strong point of this study was its high external validity for the target population because our dataset included all patients with IV CRC, regardless of treatment status.

This study has several limitations. First, our results were based on observational epidemiological inferences, and we could not examine genetic and epigenetic statuses, such as dMMR/MSI and *BRAF* mutations, which may provide a plausible explanation for our results. In the future, we would like to investigate the association between histological subtypes and the genetic and epigenetic statuses in stage IV CRC by focusing on Por/Muc/Sig. Second, we did not adjust for the severity of peritoneal dissemination. The Japanese Classification of Colorectal Carcinoma divides peritoneal dissemination into P1, P2, and P3 according to severity [9], but we did not distinguish between them in this study. The severity of peritoneal dissemination may be a potential confounding factor. Third, programmed death 1 (PD-1) blockade has been covered by insurance since December 2018 in Japan and is currently recommended after first-line therapy in patients with high MSI in the Japanese Society for Cancer of the Colon and Rectum guidelines [26]. However, PD-1 blockade was not used as a standard therapy during the study period in Japan. This could have led to the poor prognosis in the Por/Muc/Sig group. Fourth, we did not have the details of the complex comorbidities in this cohort. Therefore, we used CCI to assess patients’ comorbidities at first admission for CRC-related admission and adjust for comorbidities.

## Conclusions

In the present study, the Por/Muc/Sig histological subtype could be an independent prognostic factor for poor prognosis in patients with stage IV CRC. In addition, the histological subtype was associated with metastatic patterns. These findings suggest that histological subtype may be useful for predicting the prognosis of patients with stage IV CRC and for designing the treatment strategy.
